# The Hospital Nursing Supplement

**Published:** 1893-08-19

**Authors:** 


### "The Hospital" Nursing Mirror.

Being the Extra Nursing Supplement of "The Hospital" Newspaper.

[Contributions for this Supplement should be addressed to the Editor, The Hospital, 428, Strand, London, W.C., and should have the word "Nursing" plainly written in left-hand top corner of the envelope.]

### News from the Nursing World.

### ST. LUKE'S HOUSE.

A new Home for Incurable Patients of Both Sexes has been opened under this title at 50, Osnaburgh Street, near to Portland Road Station. It admits persons of every religious denomination, or of none, provided only that they are poor and are also suitable inmates for St. Luke's House. When they are ineligible for admission at general hospitals they are admitted to this home, where they can get the care and attention which is impossible amongst their own relatives. As long as a bed remains unoccupied any suitable candidate has a chance of admission if approved of by Mr. Howard Barrett, the medical director. The house is convenient, cheerful, and airy, and the rooms make very good wards. The nurses will have separate rooms, and the one devoted to the nurse on night duty has been specially selected for its quiet position in the house. Gifts of linen have been received from Irish friends of the matron, Miss Patric, and valuable offerings have come from Bolton, where she is equally well known. The sanitary arrangements have been excellently thought out, and those interested in this home for the incurable, the poor, and the friendless, cannot do better than visit it and judge for themselves of the arrangements. All the beds are free, and therefore donations will always be welcomed, whether in money or kind.

### A NEW OPENING.

A new field of usefulness seems likely to spread out before those who are willing and able to prepare themselves as women inspectors. As an attractive new departure it claims the attention of many, yet but few candidates will probably prove to possess the necessary qualifications for success. The originators of these new schemes will aim at securing picked women, those fitted in all respects for an important and responsible kind of work, and it is useless for inadequately prepared nurses to imagine for a moment that posts can be had on application. The work demands special qualifications as well as intelligent study, and private coaching will be necessary to pass the somewhat severe examinations of the Sanitary Institute or of the British Institute of Public Health. Even examinations successfully passed will not make a woman into a fully-fledged and competent inspector, and candidates will need to take up very seriously the preparation for a career of remarkable usefulness and interest.

### "THE HOSPITAL" COMPETITION FOR NURSES' HOMES.

This competition is exciting a good deal of interest in the schools, and those of our readers who desire to see the conditions, and to understand its object, should refer to The Hospital for July 15th, page clvii., and for July 29th, page clxxvii. It is attracting much notice in various countries, and we should be glad to receive further correspondence or inquiries in regard to it on any point affecting the competition. Competitors must send their names and addresses, by November 1st next, to Dr. Billings, United States Army, Washington, U.S.A., plans and papers being sent in to Dr. Billings not later than January 1st, 1894.

### WITHOUT A NAME.

A nurse who says she has a grievance writes us a lengthy letter about it. She is not working in a hospital, but in another kind of institution, nominally a charitable one, and she requests the editor to publish her details of her own personal grievances, in her own words but without any signature. In fact she wishes to give the general public the privilege of reading the productions of yet another anonymous grumbler. To this and to all similar proposals, the best answer is a brief one, and it consists in a piece of advice. Let the person who is the proud possessor of a real grievance, make a formal complaint, and lay the whole matter before the officials who are responsible for the government of the Home or Institute. If this be done in a temperate manner, and in an unselfish spirit, it will receive more attention, and carry far greater weight than any ill-advised and unsigned contribution to a public paper.

### AN AMPLE RECOGNITION.

Nurse Chapman, who has worked as parish nurse at Windsor for ten years, received an illuminated address and thirty guineas from the inhabitants of Windsor on quitting her post. The vicar and trustees appear not to have been satisfied that Nurse Chapman attended her cases with sufficient zeal, but that a large number of parishioners were of another opinion is evident by their efforts on her behalf, resulting in the handsome donation.

### NURSING AT WINCHESTER.

We are glad to learn that much trouble is being taken at the Royal Hants County Hospital to provide the nurses with sound training and stimulate their efforts by awarding prizes for efficiency. During the year Mr. Langdon, surgeon to the hospital, has held courses of lectures, followed by examinations, and this theoretical portion of the training and that received in the wards has been most usefully supplemented by classes for technical instruction in cookery. These, we are glad to learn, have been well attended and much appreciated. It is becoming universally admitted that no course of training for a nurse is complete without a knowledge of sick-room cookery. Our cousins over the water have learnt their lessons well, and the diet kitchen at the Johns Hopkins Hospital, Baltimore, which serves as a cookery school for the nurses, is a development that any nurse training school may be proud of. Illustrations of the complete little kitchen and articles on the subject appeared in our columns in the issues for July 16th and July 23rd, 1892.

### STOUT IN PROPORTION.

It is well that tedious business should be relieved by some amusing incidents. The Boston Board of

Guardians in the course of filling the vacant post of assistant nurse received an application from a candidate who described herself as "just turned twenty-one, five feet seven inches high, and stout in proportion." In spite of this candid description, which caused some amusement to the Board, another candidate was elected.

### THE CONTROL OF NURSING.

Dr. Groves, of Carisbrooke, Isle of Wight, at the meeting of the British Medical Association at Newcastle, brought up the subject of the control of nurses by the medical profession, which was much discussed at the meeting of 1891, the outcome of which had been, it is stated, a libel action. Dr. Groves on this occasion asked that the resolution be affirmed. Dr. Cousins said the subject was under the consideration of the council, but that the resolution which ran that "the nurses of every denomination were to be under the complete control of the profession" was not likely to have a practical outcome. Dr. Groves, like, possibly, a fair number of medical men, has found the district nurse especially, somewhat inclined to usurp his authority, but we hope that it will never be necessary to resort to stringent regulations, which would be difficult of adoption in practice, and slighting to the common sense which, we hope, superior training is increasing—not decreasing—amongst nurses.

### ILLINOIS TRAINING SCHOOL, CHICAGO.

We are glad to announce that Miss L. L. Dock, who has held the post of Assistant Superintendent at the Johns Hopkins Hospital, where she won a high reputation, has been appointed Superintendent of this School. Miss Dock belongs to the advanced school of working women, and advocates absolute independence for herself and her co-workers. She is fearless, capable, and endued with an excellent spirit, qualities which will be put to the severest test when she comes to grapple with the medical negligence and abuses which have given the Cook's County Hospital such an unenviable reputation. We believe that no wiser choice could have been made, and we cordially congratulate Miss Dock and the Johns Hopkins Training School upon the honour which this appointment undoubtedly confers.

### OTHER AMERICAN NEWS.

Miss Darche, Superintendent of the New York City Training School for Nurses, is now in this country, and we bespeak for her a hearty welcome at the hands of the matrons and superintendents of our leading hospitals. Miss Darche is travelling with Miss Kimber, the Assistant Superintendent of the New York City Training School for Nurses, and as she controls one of the leading American schools, and is besides a lady of trained intelligence and capacity, we hope her visit may prove as pleasant to herself as it will undoubtedly be interesting to all who may have the pleasure of meeting Miss Darche in England.—Miss M. A. Nutting, who has been trained at the Johns Hopkins Hospital, has been appointed to succeed Miss Dock as Assistant Superintendent there.—Miss Boland, whose book on invalid cooking was favourably reviewed in The Hospital for March 25th, 1893, is leaving the Johns Hopkins Hospital, where she has done much excellent work, in order to extend a sound knowledge of invalid cookery amongst the nurses' training schools and other institutions of the United States.

### TO BE LEFT TILL CALLED FOR.

The practical department of the Fitch Crêche and Training School for Nursery Maids at the World's Fair might well be named the Parents' Assistant, for it takes charge of the children of visitors. There is accommodation for ninety little ones, and ten nurses are in constant attendance. Every morning children between the age of four weeks and six years are received for a payment of twenty-five cents. The parents receive a brass check, and go away contented to know that their offspring will be fed, washed, and looked after generally until they fetch them away. We may be forgiven for wondering why such young creatures should be exposed to the heat and fatigue of the exhibition, and we have an old-fashioned notion that babies are safer in their own nurseries than when they are concentrated in masses. Still, the crêche is of great benefit for poorer mothers who neither attain to exhibitions nor nursemaids, and doubtless the model cribs, bassinettes, and swings form a pretty show when they are furnished with living occupants.

### THOROUGHNESS.

To begin at the beginning is really not so easy as it sounds, and most of us attempt to start half way down the course which leads to the goal of our ambition. Even the child attempts to build castles and to paint landscapes before he knows how to lay bricks or to mix colours. The girl's first attempts at cooking are centred on an elaborate sweet dish or an entrée, and she disdains to find out the best methods of roasting or boiling meat. In nursing, the setting of broken bones or the picturesque bandage attract a probationer's thoughts, while she condemns as uninteresting the making of beds or sponging of patients. Perhaps classes in "First Aid" unconsciously tend to delude the uninitiated. Everything done by the lecturer looks so pretty and so easy, and the certificates are such tangible rewards for a limited amount of attention. But building, painting, nursing—whatever we want to do, must have a solid foundation if our work is to be lasting. We may achieve a momentary success by "beginning in the middle," but everything of permanent value has to be taken up conscientiously from first to last.

### SHORT ITEMS.

The Hollond Institute, Nice, is establishing a branch in Paris.—An East-end coroner recently demanded at an inquest whether the nurses who had been called as witnesses had received their fees. Hearing they had not, he remarked, "They should have been paid, for nurses need their fees even more than doctors."—The Grimsby district nurses have been busy during the past month, in spite of fine weather. Their visits numbered 502. At Edinburgh as many as 3,805 visits were paid during the same period.—Nurse Russell, who has been nurse at the Old Windsor Workhouse for seven years, on resigning her post received a testimonial in recognition of her highly-valued services.—A characteristic article on the Princess Christian appears in the *Queen* for August 5th.—Mrs. Walter Arnold, of Hadleigh, intends to hold a bazaar for the purpose of procuring funds to provide a parish nurse for Hadleigh.

### Training Schools in America.

By Miss Irene Sutliffe, Superintendent New York Hospital School for Nurses.

In the many directions in which American women have made rapid progress in the past quarter of a century, of none can they be more justly proud than of the training schools for nurses.

It is both interesting and instructive to those engaged in the work to look back upon the condition of our large city hospitals before training schools were established, and upon the struggles of the pioneer schools. To quote from a description given by one whose efforts in organizing one of the most successful schools in this city (Chicago) are well known and appreciated. "We talked with many physicians, only a few of whom sympathised with us. We were met with such statements as this: 'Oh, yes; but I know an old woman who has nursed me for years, who beats any trained nurse.' In spite, however, of every opposition from institutions, physicians, and politicians we succeeded, after meetings, speeches, &c., in getting a few influential men to rally around us, and determined to make an effort to secure money for the experiment. This was done by personal solicitation from office to office. It was a most repulsive task for refined, dignified women to undertake, but there seemed to be no alternative, and not one flinched, but met it bravely and successfully."

It is true that in many institutions the nursing was done by sisters of various religious orders, whose tenderness and sympathy were much appreciated, and whose gentle influence and holy lives must have made lasting impressions upon many, but the nursing skill was lacking, and in many cases the vows taken by those noble women greatly limited their sphere of usefulness as nurses.

It is to the women of America that we owe our pioneer schools, as well as so many other good works, and it is certainly very fitting that they should be the first to recognise the importance of so great a work for women, for in its scope of usefulness to humanity at large there is no work equal to it. These schools were so well organised that not only do many essential features remain unchanged up to the present day, but they have been followed up by other schools throughout the country.

It is impossible to get reliable statistics of the training schools of this country. As early as 1798 (thirty years before Elizabeth Fry gave instructions to nurses in Guy's Hospital, and thirty-six years before Pastor Fliedner founded at Kaiserworth the Institute for Deaconesses, for the training of women to be nurses), Dr. Valentine Seaman gave a systematic course of instruction to the nurses of the New York Hospital. In the Administration Building of that hospital, underneath his portrait, is a letter of presentation, from which the following is quoted:—

"In 1798 he organized in the New York Hospital the first regular training school for nurses, from which other schools have since been established, extending their blessings throughout the community."

From 1861 a system of regular instruction has been given at the Woman's Hospital, Philadelphia, followed by examinations and the awarding of certificates. The first recognized schools of which I can get information are the New England in Boston and the Woman's in Philadelphia. These were organized in 1872. That they are not better known is probably due to the fact that they are connected with small hospitals, limited to women and children. In 1873 three schools were established, Bellevue in New York, the Massachusetts General in Boston, and the Connecticut in New Haven. These three schools have done continuously good work. Bellevue has trained more nurses than any other school (424). In 1875 the school on Blackwell's Island (New York City) was organized under the Department of Charities and Correction. The good this school has accomplished in Charity Hospital may be estimated by comparing one of the reports of 1874 with a recent one.

To quote: "In the fever ward (40 beds) the only nurse was a woman from the workhouse under a six months' sentence for drunkenness, who told the patients without any sign of shame the story of a most shameful life. There were no chairs with backs in the hospital; round wooden benches were the only seats and the only pillow one of chopped straw. In the fever ward the only bathing conveniences consisted of one tin basin, a piece of soap, and a ragged bit of cloth passed from bed to bed." It was the opinion of the Committee that a large part of the patients in this hospital were hungry every night; butter was used occasionally, and there had been no sugar for over two months, the supply being exhausted. Order and system now reign in these wards. The patients are cared for by earnest gentlewomen, with whom we cannot associate neglect and disorder.

In 1876 the New York Hospital School was organised, and in 1877 the Buffalo General School. From this time on scarcely a year has passed without new schools in various parts of the country, a large number being in the Eastern and Middle States, but spreading to the Pacific Coast and the Gulf of Mexico. The Bureau of Education, in the Report of 1892, gives only 36 schools in the United States, and it is exceedingly difficult to estimate the number with any degree of accuracy. I have succeeded in obtaining the names of 148 schools. In these there are estimated to be 3,250 pupils, and from these schools 4,850 nurses have been graduated.

The details of government in these schools vary greatly. In most of the older schools the govenment is not vested in the hospital authorities, but a separate incorporated society has entire control of the management. In others, the school is under the same management as the hospital, and is, in fact, a department of it. While both methods have advantages, the latter seems to be growing in favour; first, perhaps, from a financial standpoint. (Many of the independent schools have no endowment, depending entirely upon contributions for support, and if these prove inadequate, which not infrequently happens, the partially-trained nurses are sent out to private cases, the income from which service is used to supplement the deficiency.) Secondly, it would seem that if the hospital and school were united, there would be less friction among the workers, as all would have a common interest, and the best work can be accomplished only when harmony and happiness prevail.

There is a unique school at Waltham, Massachusetts, which seems to show that however valuable a hospital is to a school, it is not an absolute necessity. This school was organized in 1885, and the nurses are trained by the bedsides of poor patients in their own homes. Through this school a hospital has been started; the forty-six graduates have an excellent reputation, and a number fill prominent positions.

District nursing is done by several schools; that is, nurses are sent out by the training school to care for the very poor, who, on account of family claims and other reasons, are unable to go to the hospitals. This is an excellent charity, the nurses not only caring for the sick, but teaching the principles of cleanliness, ventilation, and economy.

The Illinois School is the first, I think, to adopt the plan of providing nurses at greatly reduced rates to people unable to pay the regular charge for trained nurses. Through the generosity of a man, whose name these nurses bear, this school sends thoroughly-trained nurses for a small charge, ranging within the means of the applicant, who must give proof of inability to pay the regular charge. They are not sent to charity cases, as this branch is covered by the Visiting Nurses' Association, but to those who are able to pa a moderate sum.

Many schools are under religious influence, mostly connected with church hospitals. Of these, St. Luke seems to be the favourite patron saint. In some of these schools the religious element enters into the training. I find in a number of schools regular religious instruction and special services at convenient times for the nurses. In some the nurses are trained for missionary work, as well as to be nurses. In the missionary school at Battle Creek, Michigan, the course is five years; both men and women are trained as missionary nurses and doctors. This school is large, and the course of instruction very elaborate.

Although nursing is essentially a woman's work, still there are cases where, for obvious reasons, a man's services are required. To meet this demand a few schools for training men have been organised. The Mills School in New York was started in 1888, and its success has proved its necessity.

The Training School for Coloured Women at Hampton, Virginia, connected with the Dixie Hospital, has opened a new field of usefulness for educated coloured women among their own people.

There are several clubs and associations for nurses, among which is the Guild of St. Barnabas for Nurses, copied, together with so much that is good and useful, from our English sisters. The object of this guild is "to assist its members in realising the greatness of their calling and in maintaining a high standard of Christian life and work."

There are branches in many of our large cities, and its influence is extending. There are over 700 members in the United States.

Truly we may look back upon the work done in twenty years with some satisfaction. Our training schools compare favourably with those of other nations. The amount of good work they have done cannot be overestimated. The sick poor, as well as the rich, are cared for in the hospitals and at their homes, and each year the demands made upon the trained nurse enlarge. Asylums, schools, homes, and other institutions seek nurses as matrons and superintendents, not alone for their knowledge of nursing, but in view of the fact that their training fits them for such positions. Schools are being established all over our country, and there is reason to think that before five years more have passed there will be few hospitals of any importance in the United States without schools for nurses.

Is there not some danger in this rapid growth in number? There is no danger of the supply exceeding the demand, for the demand increases much more rapidly than the supply. The danger is in deterioration. Not all schools are well organised, too often the only object is to secure the nursing of a few patients in a most economical manner. Many hospitals have no facilities for training nurses—most of the training consisting of lectures given in the evening, when the nurses are too tired to profit by them. This will not attract desirable women who prefer the large, well organised schools. The result is young girls just out of the school-room are received, and often those whose education and intelligence are very inadequate.

We often hear of the spoiled nurse, and not with injustice. Many a nurse has left the protection of her Alma Mater with the highest aspirations and noblest aims, and all too soon these are forgotten, and she thinks more of her own comfort than of the beauty of self-sacrifice, and of the amount received for her services rather than of her resolution to give the best that she has cheerfully and ungrudgingly to her work. Why is this? May it not be due to the fact that her life is hard and that she looses sight of the ideal, and not being able to resist the pressure of outside influences, she grasps at the material reward of her labours? Can nothing be done to prevent this? Would not a well regulated association of nurses do much towards correcting these evils? With a standard so high that only intelligent and honourable well trained women will be recognised as trained nurses, and by an earnest endeavour to help and influence each other much may be done to correct these dangers and elevate the training schools of America.

### Midwives' Registration.

The following is the report of the Select Committee appointed to consider the question of the compulsory registration of midwives, which was presented to the House of Commons on the 8th inst.:—

Your Committee has sat four times and has taken most valuable and important evidence, which, with that given last year at six sittings, includes that of distinguished medical men and women in various spheres of practice both in town and country, and also from trained and experienced midwives from various districts.

Your Committee are of opinion that a large number of maternal and particularly infant deaths as well as a serious amount of suffering and permanent injury to women and children is caused from the inefficiency and want of skill of many of the women practising as midwives, without proper training and qualification. They find that amongst the poor and working-classes, both in the country and in towns, the services of properly trained midwives have been eminently successful and of great advantage to the community. As proved by the evidence before your Committee, the services of midwives are a necessity, and, consequently, every precaution should be taken to discourage the practice of women who are ignorant and unqualified.

Your Committee are of opinion that by legislative enactment no woman should be allowed to call herself or to practice as a midwife except under suitable regulations, but that the term "Registered Midwife" should be protected and restricted to those who have been properly trained, and who alone should be placed on "The Midwives' Register"; and that the vested interests of untrained midwives should be efficiently protected by inserting, in any future Bill, a clause to the effect that, any woman who produces evidence that she is in practice as a *bona-fide* midwife at the time of the passing of the Act, shall, without formal registration, be allowed to continue her calling under the term "Midwife" alone, but shall not be permitted to assume any other title whatsoever.

Your Committee therefore recommend that a system of examination and registration of midwives should be established, and that for the purpose of admission and examination of women desiring to act as midwives, the General (Medical) Council shall be invited to frame rules for regulating—

(1) The admission to the register either by (*a*) practice, or (*b*) examination, or by both;

(2) The conditions of admission to such examinaitons, and

(3) The conditions of such examinations.

These rules and regulations should, in the opinion of your Committee, be subject to confirmation by the Privy Council, and in the event of the General Council failing to make such rules as the Privy Council can confirm, your Committee recommend that the Privy Council should invite some other medical body or forthwith cause the rules and regulations proposed in the foregoing paragraph to be framed for the purposes required, and that such rules shall take effect as if they had been made by the General Council, and confirmed by the Privy Council.

Your Committee also recommend that the duty of carrying out locally the provisions of the Act that will be required should be placed in the hands of the county councils. They also are of opinion that greater facilities for the study of midwifery should be provided in workhouse and lying-in hospitals.

In conclusion, your Committee desire to refer to the apprehension expressed by certain witnesses belonging to the medical profession, lest their interests might be injuriously affected by an improvement in the status of midwives. The great preponderance, however, of medical and other evidence, having regard to both the authority and number of the witnesses, was to a contrary effect. Your Committee, therefore, whilst giving due consideration to the expression of such fears, believe that the suggested injury is not likely to prove serious, and they are of opinion that medical men will not only be relieved of much irksome and ill-paid work, but also that improved knowledge on the part of midwives will induce them to avail themselves more frequently, and at an earlier stage than at present of skilled medical assistance in time of emergency and danger. On this point your Committee had full and substantial evidence.

### Nurses' Homes.

### I—PAST.

A FEW years ago the nurse who left the safe but expensive shelter of an institution and determined to set up for herself, had a somewhat hard struggle before her. The prejudices against women living alone free from the protection of family life were great, and the right of one half of humanity to follow an independent healthy life, with room for self-development had yet to be recognised. The nurse working for herself had often to wait long between her cases, and the struggle to make both ends meet was consequently a hard one. Necessities were brought down to the lowest limits, and comforts and refinements were dispensed with.

A small unfurnished room was taken in a back street at a weekly rent of three to four shillings, or perhaps two or more adventurous spirits shared a better home amongst them. No attendance could be obtained, and the tired nurses returned home to light their own fires, &c. Whilst waiting for her next case, a nurse was often afraid to go out lest the landlady should neglect to answer the bell and thus an engagement might be missed.

But it was in the matter of food that such nurses economised most. Bread and butter, and tea, tinned meats or occasionally something from the cook-shop formed the staple diet while not at work. Those with strong digestive organs and a healthy appetite for bread and cheese fared the best, but between cases, a wholesome nourishing meal was, for sufficient reasons, rarely obtained.

Yet it must be remembered that these women were, physically, of sterner stuff than those of the present day, They came of a different class, and were used to rough work and plain living. More passive of temperament, and therefore less despondent, they could wait patiently for long intervals, and did not suffer from the mental worry of inactivity. Accustomed to a low standard of wages for women, they took any fee that offered, however small, and trusted to an occasional rich patient to make up the deficiency in the payments of the poor. Their scientific training was limited, but they did their work conscientiously and well, rarely leaving their patients, and with few expectations of daily outings on evenings off duty. Their pleasures were simple, and their world was contained in the narrow limits of the sick-room, and the servants' hall, in which they took their meals and spent their spare moments. The troubles and joys of their comrades in the kitchen formed the chief topics of conversation and sometimes served to amuse future patients who expected nothing higher from them. Usually it was a kindly interest in the welfare of the household that gave speed to the nurse's tongue, and her departure was attended with mutual promises of meetings and tea drinkings later on. Sometimes she proved a mischief-maker and scandal-monger and her departure relieved everyone from a heavy burden. The low standard of living and the few pleasures of this class of nurse, rendered her peculiarly liable to the abuse of stimulants, and her alcoholic breath too often poisoned the atmosphere of the sick-room and disgusted her patient. This explains the unpleasant clause concerning beer money, still mentioned on the papers of the older institutions originally formulated as an incitement to temperance on the part of the nurse.

Such a woman required few of the home comforts insisted on by her sister of to-day. Her little attic crowded up by the big bed, and adorned with one or two inartistic prints and faded portraits was in no sense a home, but a shelter in which to wait for work. To buy books and flowers and such luxuries she deem wasted money. The old-fashioned nurse, if temperate, was saving every penny for a blissful future when the small sums would stock a little shop or a house to let out in apartments.

At institutions the nurses then fared little better than when working for themselves. A back room looking on a blank wall or an underground kitchen, was, as a rule, the only apartment provided for recreation and meals. A table covered with a stained cloth occupied the centre of the floor, and hard Windsor chairs with sometimes the added luxury of a horse-hair couch made up the furniture of the dismal room.

Its adjornment usually began and ended in coloured prints of the Royal family, and a few well-thumbed novels which, with one or two works on religion, formed the entire library of the home. Nor was the sleeping accommodation any better. Two nurses often occupied the same bed, and shared the same toilet accessories, until quite lately separate arrangements for washing and dressing were unknown in the private nursing homes, and screens and curtains were conspicuous by their absence. Two and even three bedsteads were crowded into one room, and the cleanliness of blankets, beds, and pillows left much to be desired. The nurse was expected to be down to breakfast at eight punctually, and only in extreme cases allowed to stay in bed the first morning after her return from a heavy case. Unless special leave was given she was expected to be in strictly at ten p.m. This permission was rarely refused, but it was granted as a favour, and a nurse requesting it too often was looked on with suspicion. On the other hand some of the institutions were in the hands of homely people, who treated the nurses in every way as belonging to their own family, and who laid little restriction on their freedom when disengaged. The meals at these places were usually fairly good in quality and quantity, but badly cooked and indifferently served. In all cases the slip-shod mis-management of the past forms a strong contrast to the business-like method and carefulness of detail which obtains in the private nursing home of the present day.

### Everybody's Opinion.

[Correspondence on all subjects is invited, but we cannot in any way be responsible for the opinions expressed by our correspondents. No communications can be entertained if the name and address of the correspondent is not given, or unless one side of the paper only be written on.]

### AUSTRALIA OR NEW ZEALAND?

"SISTER CECILE" writes: In reply to an enquiry made by "Sister Y." in THE HOSPITAL, I beg to suggest that Wellington, Martow, and Wanganui, in New Zealand, are all fairly good places for consumptive patients. In all these towns good medical advice can be secured. A large supply of clothes is desirable, including thick and thin woollen garments, muslins, and prints.

### ENGLISH NURSES IN PARIS.

"SISTER A." writes: Having recently returned from nursing in Paris, I am anxious to make known the need there appears to be for a good private nursing institution, one conducted on the co-operative system—and as Paris is an expensive place to live in, a home where the nurses would be made comfortable. From observations made while in Paris, I think that now is the time to start such an institution ready for the coming winter season. There are always a great many English and Americans staying in Paris, as well as the usual residents, and from what I have heard, the English doctors would welcome an association which would supply fully-trained nurses. This scheme, of course, needs capital and some influential support, but if started and worked on good business principles, should be a success.

I trust that someone may be found through the publicity of The Hospital willing to take up this scheme, or to suggest some method by which such an undertaking could be commenced.

*** We beg to draw "Sister A's" attention to the announcement respecting the Hollond Institution in "Short Items.—Ed. *T.H.*"

RATING HER SISTERS.

"One Who is Proud of Her Hospital" writes: With reference to the recent attack on the London may I express my opinion on the subject? The time I spent there was one of the happiest I have spent during my nursing career (ten years). I (and I am sure many others must) feel more than disgusted when I hear constant discussions as to what nurses eat, drink, and wear. Is this their sole object in life? If only they would pay a little more attention to the simple every day rules of cleanliness both in work and person we should find less dirt and filth (I speak advisedly) in some of our infirmaries and school hospitals; but their person seems their first and sole consideration, and patients must do the best they can. There is no doubt a great number of ladies "go in for" nursing through some great disappointment in life, and then when they find they have something to do besides smoothing pillows and reading tracts, pose as martyrs and try to bring discredit on our noble institutions. I think both sisters and charge nurses have a great deal to put up with from probationers; I know they had with me, although I tried my best to do right. In conclusion, I must say that I shall never forget all my good training, neither can I ever express my gratitude and thankfulness to those who bore with me so patiently. That the institution and its Matron may always prosper is my heartfelt wish.

OUTDOOR UNIFORM.

"Only a Pro" writes: I have been much interested in the above discussion, and surprised that a nurse should raise a question on such a subject. It is admitted by all that exceptional qualifications are necessary for a successful nursing career, such as self-forgetfulness, gentleness, patience, forethought, &c. Very well, then, if this is needed to make a thorough nurse, she must be a woman different to the ordinary everyday class one meets, and thus, in keeping with the rest of her life (for it is a life of hard work and incessant self-denial), a distinct costume is not only desirable, but her due—a costume not constituted to make her conspicuous or draw forth remark and condemnation from the public, but one which will allow her to appear as neat, tidy, and self-composed out of doors as she should do in, as well as gain for her the respect the public are so willing and ever ready to give a true nurse.

THE CAPE MISSION.

**"K. R. W." writes: I believe the Cape General Mission, 20, The Paragon, Streatham Hill, S.W., send out nurses to South Africa. I do not think it would be a good place for Nurse Edith to go out on her own account, unless she has an income to fall back upon, as everything is very expensive in South Africa, and some time must necessarily elapse before she becomes sufficiently known to depend on her work. If Nurse Edith applies to the above address she will obtain information concerning qualifications, &c., necessary.**

DUBLIN.

**The Private Nurses' Association in Dublin has done well since it was first started a year ago. It has at present about thirty-five members, many of whom are non-resident. Of course the nurses who board at the Asso ciation get most employment, being on the spot in cases of emergency, but they were all in constant work all last winter, and two or three have found permanent situations. None but fully trained nurses are eligible, and each nurs- must bring a testimonial from a lady, preferably from the Superintendent of nurses at the hospital where she was trained, as to her moral character and sobriety, as well as from doctors for whom she has worked. Nurses passing through Dublin will like to hear that they can use the Association as a hotel at very reasonable rates; if subscribers to the Association they will be on the same footing as resident nurses, and consequently be accommodated at a cheaper rate than non-subscribers. They are requested to write to the Matron, Private Nurses' Association, a day before their pro posed arrival.**

GIRL NURSES.

**"A Girl Nurse writes: Having read an article in a recent issue of The Hospital entitled "More Girl Nurses," I should like to offer my humble opinion on the subject. Speaking from personal experience, I began at the age of 18, have been nursing for over five years, and now at the age of 23, am exceedingly glad that I did not have to wait till I reached the "mature age of 25," but that the lady superintendent of the charming hospital where I was trained, and to which I am proud to say I still belong, much prefers young nurses to those of mature age. I have seen many nurses during these five years, and with very few exceptions, the most successful in their work, the most obedient to orders and the smartest in the fulfilling of them were the young nurses. Some people hold up the plea of injury to health as a reason for girls not entering the wards before 25. Speaking again from personal experience, I think that girls are just as strong and fit for hospital work at 18 or 20 as they will be if they sit down at home and vegetate till they are "matured" enough to begin, or else spend the time of waiting in tennis parties, dances, &c., which only unfit them for the work they are about to undertake. For my own part, I can say I never felt better than since I have been in hospital, and I know there are many other nurses of my own age, both here and in other hospitals, who will agree with me. Of course, there are exceptions to every rule, and many girls are prevented by home duties, &c., from taking up hospital work so early; and again, many girls of 20 are quite unsuitable for hospital work from various reasons, either from flightiness of character, untruthfulness, &c. But it seems to me that these undesirable qualities will not grow less with increasing years, and those who are from such reasons unfit for hospital work at 20, will be, if anything, more unfit for it at 25.**

### Appointments.

[It is requested that successful candidates will send a copy of their applications and testimonials, with date of election, to The Editor, The Lodge, Porchester Square, W.]

Lynton Cottage Hospital.—We learn that Miss May Kenrick has lately been appointed to the position of Matron of the Lynton District Cottage Hospital, at Lynton, North Devon. Miss Kenrick was trained at the London Temperance Hospital, and was afterwards staff nurse at the Ilfracombe Cottage Hospital.

Clun Cottage Hospital.—Miss Constance Chambers has been appointed Matron of the St. Catherine's Cottage Hospital, Clun. Miss Chambers was trained at the Salisbury Infirmary, and was afterwards nurse at the Warminster Cottage Hospital, and later district nurse at Maidenhead. There were fifty applicants for the post which Miss Chambers has obtained at Clun.

Warminster Cottage Hospital.—Miss M. Wilks has been appointed Matron of this hospital. Miss Wilks trained at Salisbury, and has for nearly two years been charge nurse of the Male Medical and Surgical Wards at the King's Lynn Hospital, where she has gained the love and respect of both the Lady Superintendent and nurses, and has proved herself an excellent and conscientious nurse.

### The World of Nurses.

As seen by an Unprofessional Correspondent.

### I.—DETERIORATION.

It has long been a sorrow to many of us, to see the lowering of tone amongst nurses of all kinds. Long ago, it was said that women only took to the profession in order to find husbands, and unfortunately, we all of us discovered against our will, much corroboration of this assertion. But, on the other hand, we have, most of us, known splendid examples to the contrary—women whose aim in life was to do good. Still the fact remains, that the general tone of the nursing profession has lowered, as the numbers have augmented. As to their professional capabilities, I am not fitted to pass an opinion; it is of the moral aspect that I would speak.

Years ago, when the first movement was made by the establishment of St. John's House, to supply a thoroughly efficient class of nurses for the sick poor, devoted women were found to undertake the work for sheer love of doing good. Florence Nightingale established her society upon the basis of good work demanding monetary payment. The sisterhood idea, carried out by the sisters of All Saints, of East Grinstead, of Clewer and others, and in a lesser degree by those of St. John's House, was not agreeable to Miss Nightingale's views; but at the same time, a memorandum drawn up by her, or at her request, for use at St. Thomas's Training School for Nurses, shows that her main idea as to the proper qualification for a hospital nurse was "self-sacrifice." "We want women who are conscious that they were sent into the world for something more than the pursuit of their own gratification, and who feel life is not worth living unless they strive to make the world something better for their having lived in it. . . . They must be prepared to make sacrifices, without thinking themselves self-sacrificing." Such was the class of women who formerly devoted themselves to nursing. The pay, if any, was small; but love filled up the purse; "to do good," was the main object of those women. The object now, it is feared, is a far lower one—that of getting a living in a genteeler walk than domestic service; and not infrequently, the catching of a husband. If this be otherwise, how comes it that so many nurses marry doctors, often students at their own hospital? How is it that one meets a student and a nurse (to use a mild term) talking earnestly in the corridors? How comes it that one sees nurses in uniform at theatres, shows, entertainments of all kinds, with, what to most of us appear to be, very undesirable companions?

Then, again, look at the style of the present nurses—much white collar and cuffs. When we *mondaines* cover up our cuffs and collars with our cloaks and jackets, the nurse displays hers as much as possible; so, too, the white strings and apron. Why is it necessary to go out for a walk in an apron? Formerly the object of a uniform was to protect the wearer from insult, now it is a means of attraction. One sees it everywhere, from the Burlington Arcade to Regent Street, and such like resorts, in all its attractive aggressiveness, and accompanied by fringes and furze-bush hair, high heels, and ringed, gloveless hands. It meets us in the omnibuses, and its owners talk "shop" in a voice intended for everyone to hear. For one nurse in the streets twenty—even ten—years ago, you meet a hundred now—red, blue, green, grey, brown, and black.

But I am told many of these women are not nurses, and if so, is it not sufficient reason for abolishing once and for ever all out-door uniform. Of course I know the plea for not changing dress—the fatigue. But it is more cleanly to do so, and safer as regards infection.

But what is the reason of the decadence? Is it not, perhaps, the fact that by giving poor pay for such work you get an inferior class of workers? If the calling be one of love only you get a devoted class of women who are ready to sacrifice themselves; but if you give less wages than in other walks of life, you get a class of persons who are too ignorant or too silly to earn a living otherwise. £20 to £25 a year is small pay for an educated woman; £70 to £100 is a miserable dole to one who is qualified to fill the post of matron at a hospital where the resident medical officers, the chaplain, the steward, and other masculine officials have twice, thrice, and four times as much. A nurse ought either to be paid efficiently, or her services ought to be a labour of love. We do not find teachers and governesses marrying their professors; now and then one, doubtless, but not to the same degree. Moreover, as a proof that marriage is one of the ends of nursing life, you need only turn over the pages of the *Matrimonial News*, where you may see advertisements headed "Hospital Nurse." If this be genuine, it is a disgrace to the profession; if it be a bogus advertisement, its mere insertion proves that nurses are amongst readers of the paper.

Cannot something be done? Will not matrons and committees lay this matter to heart, and, in the first place, abolish all outdoor uniforms? Surely a more careful choice might be exercised; surely matrons might dismiss at once any nurse found upon a corridor with a student; surely more strict rules as regards display of white adornments, of fringes, and high heels might be instituted. The illustrated advertisements of nurse costumes—veils which are worn down the back for no purpose but pictorial effect, curls and noiseless heels, skittish caps and bonnets, &c., are quite sufficient proof that my censure is not too severe. It is a grievous thing that the chance of helping some poor waif or stray in hospital should be lost because a nurse is frivolous, vain, and worldly-minded. Endless are their opportunities of helping sick souls as well as tending broken bodies; but if the nurse's one idea is the embellishment of her own person, she certainly will give small thought to her patients.

The nurse's calling is the noblest to which a woman can devote herself, if she does so modestly and fearlessly, as unto God; but if the motive be the advancement of her own vain-glory and worldly position, she degrades herself and her work.

Aihpos.

### Death in Our Ranks.

City of London Lying-in Hospital.—In a previous issue we reported the resignation of Miss S. E. Allen, the Matron of the City of London Lying-in Hospital, in consequence of ill-health, we now regret to state that she passed away on the 11th inst., at Hampstead after a long and painful illness. Miss Allen was a devoted worker, and the great work she did at this hospital will never be forgotten. She was buried at Highgate Cemetery on Monday, and her funeral was attended by a large number of old patients and pupils who had good cause to testify their gratitude to one who was deservedly and much beloved by all classes. We cannot adequately express the very sincere regret which the Committee and officers feel at the irreparable loss the hospital has sustained by Miss Allen's death.

### Notes and Queries.

### Queries.

(171) S. E. B. (*Ambulance*).—You do not observe our rules, and give no name or address.

(172) *L. C. A.* (*Italy*).—I am anxious to go to Italy for the winter months as private nurse. Can you put me in the way of doing so?

(173) "*The Nurses' Home" Competition.*—Could you tell me when the competitors are to send in their plans for Nurses' Home, and where?

(174) *W.*—Will you give me name and address of the head of the North London District Nursing Association?

### Answers.

(172) *L. C. A.* (*Italy*).—Write to the Directress of the Hollond Institution, All Saints' Rectory, Axminster. The Lady Superintendent St Paul's House Nursing Institution, Rome, and we also advise you to advertise.

(173) "*The Nurses' Home" Competition.*—See statement in "News from Nursing World" this week's issue.

(174) *W.*—Miss de Luttichau, Lady Superintendent, 413, Holloway Road; also the Hampstead Home Hospital, and many other institutions are mentioned in "Burdett's Hospital Annual." The Scientific Press, 428, Strand, W.C.

### The Muses' Looking Glass.

### MEDIEVAL LORE.*

A good many persons have been concerned in the making of this very entertaining and instructive little volume. We have first the author, the English Franciscan monk Bartholomew, who about the year 1260 compiled in Latin what he termed an "Encyclopædia on the Properties of Things." Next there comes the translator; the version quoted "was made for Sir Thomas, lord of Berkeley, in 1397, by John Trevisa his chaplain." John Trevisa seems to have fared badly at the hands of his editors, and Berthelet, who in 1535 brought out the edition from which this volume is compiled, cut down his author right editorially. The present editor, Mr. Steele, claims "the privilege of his predecessors" so far as to alter the spelling and sometimes the grammar of his author "in making a selection of passages from the work for modern readers"; he is perhaps as much entitled to assume this liberty as his readers are to grumble at what will appear to many of them a serious detraction from the value of the transcript. Lastly, we have a characteristic preface from Mr. William Morris, pointing out the charms of Middle Age science, and calling attention to the fact that "the people of that time were eagerly desirous for knowledge, and their teachers were mostly single-hearted and intelligent men, of a diligence and laboriousness almost past belief."

After a good proportion of introductory matter has been got through, the book is found to consist of an epitome of mediæval science, manners, medicine, geography, and natural history. The extracts have wisely been chosen with a view to illustrating the immense importance of the book to Elizabethan literature. Incidentally, they serve to throw light on all the literature of the age, and it is earnestly to be hoped that the editor may be sufficiently encouraged by the reception accorded to his labours, to offer the public the work in a complete and unmodernized version. A knowledge of mediæval science is absolutely essential to the right understanding of many authors, and in ancient astronomy in particular (from which, unhappily, no extracts are here given), the ignorance of the modern student of literature makes many passages of the poets a complete enigma. Of mediæval science, indeed, there is altogether far too little. We quote the passage relating to the sapphire as illustrating what Mr. Morris calls "a certain quaint floweriness in the author."

"The sapphire is a precious stone, and is blue in colour, most like to heaven in fair weather and clear" (compare Dante's description of the sky as he emerged from the Inferno, "Dolce color d'oriental zaffiro"), "and is best among precious stones most apt and able to fingers of kings. Its virtue is contrary to venom, and quencheth it every deal. And if thou put an addercop in a box, and hold a very sapphire of lux at the mouth of the box any while, by virtue thereof the addercop is overcome and dieth, as it were suddenly, and this same I have seen proved oft in many and divers places."

The extracts relating to medicine contain many curious dicta, and the treatment of frenzy or "woodness" is specially quaint, though less barbarous than might be expected. "The diet shall be full scarce, as crumbs of bread, which must many times be wet in water. The medicine is, that in the beginning the patient's head be shaven, and washed in lukewarm vinegar, and that he be well kept or bound in a dark place. Diverse shapes of faces and semblance of painting shall not be showed tofore him, lest he be tarred with woodness. All that be about him shall be commanded to be still and in silence; men shall not answer to his nice words. In the beginning of medecine he shall be let blood in a vein of the forehead, and bled as much as will fill an egg-shell. The head that is shaven shall be plastered with lungs of a swine, or of a wether, or of a sheep; the temples and forehead shall be anointed with the juice of lettuce or of poppy. If after these medecines are laid thus to, the woodness dureth three days without sleep, there is no hope of recovery."

The description of a mediæval physician is well worth citing since these worthies did not always come off very well at the hands of their contemporaries. "A physician visiteth oft the houses and countris of sick men. And seeketh and searcheth the causes and circumstances of the sicknesses, and arrayeth and bringeth with him divers and contrary medecines. And he refuseth not to grope and handle, and to wipe and cleanse wounds of sick men. And he behovteth to all men hope and trust of recovering of health; and saith that he will softly burn that which shall be burnt, and cut that which shall be cut. And lest the whole part should corrupt, he spareth not to burn and to cut off the part that is rotted, and if a part in the right side acheth, he spareth not to smite in the left side. A good leech leaveth not cutting or burning for weeping of the patient. And he hideth and covereth the bitterness of the medicine with some manner of sweetness. He drinketh and tasteth of the medecine, though it be bitter, that it be not against the sick man's heart; and refraineth the sick man of meat and drink; and letteth him have his own will, of the whose health is neither hope nor trust of recovering."

The natural history is full of marvels; almost every line opens up some new light on obscure passages in the poets. Relying largely on tradition, the English Bartholomew has his own individual tastes notwithstanding, and makes them felt with a vigour, which loses nothing in the translator's hands. Some of his traditions vary from those commonly received as for instance this of the pelican: "The pelican loveth too much her children. For when the children be taught and begin to wax hoar, they smite the father and the mother in the face, wherefore the mother smiteth them again and slayeth them. And the third day the mother smiteth herself in her side, that the blood runneth out, and sheddeth that hot blood on the bodies of her children. And by virtue of that blood the birds that were before dead quicken again." Such a legend illustrates perfectly the symbolism of the pelican ornamentation often found in medieval architecture, notably at Holy Cross, near Winchester. But when we come to the domestic animals Bartholomew leaves his authorities for the witness of his eyes and ears; there is suggestion of sleepless nights varied with throwing of missiles in the account of the cat. "In time of love is hard fighting for wives, and one scratcheth and rendeth the other grievously with biting and with claws. And he maketh a ruthful noise and ghastful when one proffereth to fight with another; and unneth is hurt when he is thrown down off an high place."

The geography is for the most part a mere reproduction of Pliny and noteworthy only where it touches countries which have been under the author's personal observation. The remarks on the Irish read like an amplication of certain recent Tory utterances. "The people there use to harbour no guests, they be warriors, and drink men's blood that they slay, and wash first their faces therewith: Right and unright they take for one"; while the judgment on the English as "free men of heart and with tongue, but the hand is more better and more free than the tongue," has lately met with striking confirmation in the remarkable demonstration which by universal consent is to be buried as "a regrettable incident."

* "Medieval Lore."—Edited by Robert Steele (Elliot Stock).

### Nurses in Workhouses.

With the admitted advantage of trained nurses for workhouse infirmaries arises also a necessary demand for improved accommodation and comfort for the nurses. At a recent meeting of a board of guardians it was suggested that part of a vacant ward should be partitioned off with wood to form a bed-room for a nurse. Fortunately for the nurse, should the vacant ward ever be utilised, one of the board suggested that lathe and plaster was more suitable, and it was agreed to carry his suggestion out.

### The Book World for Women and Nurses.

[We invite Correspondence, Criticism, Enquiries, and Notes on Books likely to interest Women and Nurses. Address, Editor, The Hospital (Nurses' Book World), 428, Strand, W.C.]

Beauty and Hygiene. By A Specialist. (Swan Sonnenschein and Co.)

The title of the book before us is somewhat deceptive, and will no doubt obtain an entrance for it into homes from which it will be as quickly expunged.

The word "Hygiene" catches the eye, and one expects something pure and sound in the matter of health. Nothing of the sort—the hygiene belongs to the cover, and the cover only, in this case. The contents are simply a kind of manual for the use of women and girls in the art of "making up," and very pernicious and unsanitary most of the suggestions are.

As we lay the volume down we seem all at once to have been admitted behind the curtain—to have seen the legs of the Punch and Judy man and the wires of the puppets, and all ideality for ever driven from the show.

We are glad to see that some sense of shame still exists amongst the women who practice these unholy arts, as we are told that they should be regarded as "toilet secrets," "for men do not like seeing how it is done!" Do the men, or those of them whose opinion would be of any value, like it when it is done? This may be open to doubt. Perhaps if women knew more what men say amongst themselves on these points they would not be so "cock sure" as to the measure of attractiveness they are able to command.

The receipts and appliances recommended are too many and, in some instances, too disgusting to quote, but one erroneous assertion made is that a "charming milky lotion will soften the skin and prevent wrinkles." The softening of the skin aids in producing them!

On page 60 we are told that "every well educated woman should be perfectly aware of the physiological reasons which make excessive tight-lacing a positive crime," and the next paragraph lays it down as an incontrovertible axiom that "19 or 20 inches is the smallest size permissible!" What is to be understood by the term "excessive," if seven or eight inches less than the circumference usually allowed by anatomists as the proportionate one for a woman's waist be not such?

To sum up the matter in few words, we would say that any woman educated to know the "crime" of compressing her figure to these unnatural proportions, or rather distortions, would, we should hope, find some better outcome for her mental energies than in spending valuable hours in the concoction of poultices and swathing bands, whereby to inflate her bust or in the search after an "ideal" corset by means of which to cheat nature, and give herself the regulation size for a fashionable waist.

If the book is written to meet the wants of the present time, we can only feel sorry for the age in which we live, and for the women who so degrade their womanhood as to practice the arts laid down in the pages before us. But we would hope better things than these, and believe that amongst really refined and cultured women there would be few who could read of the means and appliances to which we have briefly alluded without a sense of shame and repugnance equal to our own.

**THE BOOK WORLD FOR WOMEN AND NURSES**—*continued.*

AUGUST REVIEWS.

The old question of the comparative powers of man and woman which seems to have taken the place of the big gooseberry or the sea serpent in the journalism of to-day is approached in the New Review by Professor Büchner, under the aspect of "The Brain of Women." The contents of a good-sized coffee cup, it appears, about equal the difference in brain space of the sexes. The higher in culture the race the more perceptible the difference, which is a conclusion depressing to contemplate. The Australian, Polynesian, or Chinese woman then is more really on an equality with her lord and master than the Girton "savante" whose husband regards her attainments with a mixture of awe and annoyance. Professor Büchner will not for a moment admit anything so ungallant, but what he does admit it is not so easy to discover. He mildly observes that "daily experience teaches that woman can only with difficulty be convinced by reason when the result to be obtained is one which runs counter to her feelings," and he is inclined to believe that nature has not dealt quite fairly with the women in saddling them with the care of babies, for "the exercise of the domestic duties calls for a less active exercise of the mind than the more exacting labours of man." It would be pleasant to see the professor suddenly called on to exchange for the complex duties of the mother of a large household on small means, the "more exacting labour" of speculating on her brain powers in his study.

In "Leaves from a Note-Book," in Macmillan, there are some remarkable blunders to add to Mr. Wheatley's amusing collection. They are collected from the certificate examinations of pupil teachers, and are perpetrated by candidates aged from nineteen to twenty-one. The history papers have often a touch of the most delightful unconscious humour, as in the following: "Both the Royalist and Parliamentarian parties in the Civil War suffered from internal dissensions because their baggage being all swept away, they were pierced with cold and hunger." One young person was asked to name the chief writers of the reign of Queen Anne. This was her answer: "The reign of Queen Anne has been called the Augustan age. The chief writers of this period were Shakespeare, Chaucer, Dryden, Ben Jonson, Goldsmith, and Sir Walter Scott." Proceeding further to give an account of Shakespeare, the same examinee could find no other works for him but "To be or not to be," "The Moor of Athens," and "Dombey and Son." The writer of the article may well ask, "Are these pupils or their teachers most to blame?" On the whole, he inclines, and most people will agree with him to lay the blame on the system which demands more from both pupil and teacher than can possibly be digested. The paper of questions in animal physiology which he quotes as having been set before girls, aged from twelve to fourteen, is worth reproducing for its absurdity: (1) Write what you know of the intestines, their structure, and functions; (2) describe the pulmonary or lesser circulation; (3) explain artery, vein, oxygen, mastication, osmosis, respiration; and (4) draw a diagram of the stomach, describe its position, structure, and functions. He may well say, "When one reflects on the genius for blundering shown in such comparately simple matters as history and literature, the possibilities offered by the intricacies of the human stomach are appalling!"

